# Immunogenicity and Induction of Functional Antibodies in Rabbits Immunized with a Trivalent Typhoid-Invasive Nontyphoidal *Salmonella* Glycoconjugate Formulation

**DOI:** 10.3390/molecules23071749

**Published:** 2018-07-17

**Authors:** Scott M. Baliban, Jessica C. Allen, Brittany Curtis, Mohammed N. Amin, Andrew Lees, R. Nageswara Rao, Gangadhara Naidu, Ramasamy Venkatesan, D. Yogeswara Rao, Vadrevu Krishna Mohan, Krishna M. Ella, Myron M. Levine, Raphael Simon

**Affiliations:** 1 Center for Vaccine Development and Global Health, University of Maryland School of Medicine, Baltimore, MD 21201, USA; sbaliban@som.umaryland.edu (S.M.B.); jessica.allen@umaryland.edu (J.C.A.); bcurtis1@som.umaryland.edu (B.C.); mamin@som.umaryland.edu (M.N.A.); mlevine@som.umaryland.edu (M.M.L.); 2 Fina Biosolutions LLC, 9430 Key West Ave. Suite 200, Rockville, MD 20850, USA; alees@finabio.com; 3 Bharat Biotech International Limited, Hyderabad 500 078, Telangana, India; rnrao55@yahoo.com (R.N.R.); gangadhara1975@bharatbiotech.com (G.N.); venkatesanr@bharatbiotech.com (R.V.); yogeswar.danda@bharatbiotech.com (D.Y.R.); kmohan@bharatbiotech.com (V.K.M.); ella@bharatbiotech.com (K.M.E.)

**Keywords:** lipopolysaccharide, Vi, glycoconjugate, antibody, *Salmonella*, flagellin, vaccine, iNTS, typhoid

## Abstract

Typhoid fever due to *Salmonella* Typhi and invasive nontyphoidal *Salmonella* (iNTS) infections caused by serovars Enteritidis (SE) and Typhimurium (STm) are major pediatric health problems in sub-Saharan Africa. Typhoid has high complication rates, and iNTS infections have high case fatality rates; moreover, emerging antimicrobial resistance is diminishing treatment options. Vi capsule-based typhoid conjugate vaccine (Typbar-TCV™), licensed in India and pre-qualified by the World Health Organization, elicits durable immunity when administered to infants, but no iNTS vaccines are licensed or imminent. We have developed monovalent SE and STm glycoconjugate vaccines based on coupling lipopolysaccharide-derived core-O polysaccharide (COPS) to phase 1 flagellin protein (FliC) from the homologous serovar. Herein, we report the immunogenicity of multivalent formulations of iNTS COPS:FliC conjugates with Typbar-TCV™. Rabbits immunized with the trivalent typhoid-iNTS glycoconjugate vaccine generated high titers of serum IgG antibody to all three polysaccharide antigens for which anti-COPS IgG antibodies were directed primarily against serogroup-specific OPS epitopes. Responses to SE and STm FliC were lower relative to anti-COPS titers. Post-vaccination rabbit sera mediated bactericidal activity in-vitro, and protected mice after passive transfer against challenge with virulent SE or STm Malian blood isolates. These results support accelerated progression to clinical trials.

## 1. Introduction

The bacterial species *Salmonella enterica* encompasses more than 2500 serovariants, of which a subset are associated with human clinical infections [1]. Serovar *S*. Typhi, a human-restricted pathogen, causes typhoid fever, that is characterized by fever, abdominal discomfort and frontal headaches. In the pre-antibiotic era, the case fatality rate of typhoid fever was 10–20%. Non-typhoidal *Salmonella* (NTS) serovars generally have a broad host range, and when they cause clinical illness in humans it is usually self-limiting gastroenteritis. However, in very young infants, immunosenescent elderly and immunocompromised hosts, NTS can cause invasive clinical disease.

Both typhoid and invasive NTS (iNTS) disease occur in pediatric populations in sub-Saharan Africa, with typhoid found more commonly in pre-school and school age children and iNTS found in infants and toddlers [2]. The geographic prevalence is variable, as some regions exhibit only typhoid disease, others only iNTS, and yet others have both types of infections. Remarkably, *Salmonella* infections of one type or another have accounted for ≥33% of all bacteremic disease at most sites, with many isolates antibiotic resistant. In sub-Saharan Africa, iNTS infections are due primarily to serovars Enteritidis and Typhimurium (including the monophasic variant 1,4,[5],12:i:-), present clinically without gastroenteritis, and have case fatality rates of 12–28% [3,4,5]. 

Important differences have been found between iNTS strains circulating in sub-Saharan Africa compared to the conventional gastroenteritis NTS strains found in the USA and Europe. Genomic analyses of *S*. Typhimurium and *S*. Enteritidis revealed novel multi-locus sequence types and patterns of genetic degradation analogous to those present in typhoid and paratyphoid serovars [6,7]. Pathotypic analyses of a prototype sub-Saharan African *S*. Typhimurium blood isolate found reduced inflammation and enhanced intracellular survival in cultured cells, and the absence of diarrhea in a non-human primate model—phenotypes generally associated with *S*. Typhi [6,7,8,9]. Despite ongoing research efforts, the reservoir for iNTS infection has not yet been identified, hampering the ability to implement traditional environmental interventions for interruption of transmission [10]. While there are several types of licensed typhoid vaccines, there are no available human NTS vaccines.

**Figure 1:**
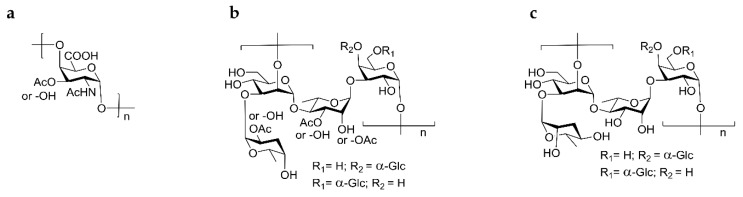
Structures of the repeat units of the trivalent *Salmonella* glycoconjugate vaccine components. (**a**) *S*. Typhi Vi capsule polysaccharide; (**b**,**c**) O polysaccharide repeats from (**b**) *S*. Typhimurium (serogroup B) and (**c**) *S*. Enteritidis (serogroup D) O polysaccharides. Adapted from references [1,14].

While they are extracellular, prior to invasion or following release from infected cells, *Salmonella* are presumed to be susceptible to antibodies that recognize bacterial surface structures. *S*. Typhi expresses a capsular polysaccharide (CPS), termed Vi, which is a homopolymer of →4)-α-d-Gal*p*2NAcA3Ac-(1→ (Figure 1a). Serum IgG directed against Vi correlates with protection against typhoid illness in humans, and unconjugated and conjugated Vi polysaccharide vaccines are licensed for protection against typhoid [11]. *S*. Typhimurium and *S*. Enteritidis do not express Vi. Hence, the surface polysaccharide in these serovars is the core and O polysaccharide (COPS) of lipopolysaccharide (LPS). These serovars share a common →2)-α-d-Man*p*-(1→4)-α-l-Rha*p*-(1→3)-α-d-Gal*p*-(1→) OPS trisaccharide backbone (serologically designated O antigen 12) and are distinguished serologically by an immunodominant α-(3→6) dideoxy hexose linked to mannose that is an abequose in *S*. Typhimurium (O antigen 4) and a tyvelose in *S*. Enteritidis (O antigen 9) (Figure 1b,c). Antibodies against NTS OPS have maintained protection in animal challenge models and thus form the basis for promising NTS vaccines [12,13].

Isolated polysaccharides from pathogenic bacteria are commonly thymus-independent antigens that fail to induce immunologic memory, class switch or affinity maturation and are poorly immunogenic in children less than 2 years of age [15]. Conjugation to a protein carrier engages T-cell help through the presentation of carrier peptides on major histocompatibility complex (MHC) molecules of antigen-presenting cells, thus overcoming the limitations of unconjugated polysaccharide vaccines such as parenteral Vi CPS [16]. Newly developed Vi conjugates are thus the first typhoid polysaccharide vaccines which are suitable for administration to children younger than 2 years old. Typbar-TCV™, comprised of *S*. Typhi Vi chemically linked to tetanus toxoid, is licensed in India and recently became the first typhoid conjugate to achieve WHO prequalification, a key step in international procurement through the Global Alliance for Vaccines and Immunization (GAVI) [17].

We previously reported the development of iNTS glycoconjugate vaccines for *S*. Enteritidis and *S*. Typhimurium, comprised of the purified COPS molecules from each serovar conjugated to the respective phase 1 flagellin protein (FliC), with the notion that FliC could act as both a carrier protein and an additional relevant vaccine antigen as anti-flagellin antibodies mediate functional bactericidal activity [18,19,20]. Mice immunized with monovalent *S*. Typhimurium or *S*. Enteritidis COPS:FliC conjugates mounted robust immune responses to both the carrier and hapten and were protected against challenge with virulent blood culture isolates of the homologous serovar [13,18,19,21]. The ultimate goal of this vaccine development effort is the production of a trivalent formulation to provide broad coverage for pediatric typhoid and iNTS infections in sub-Saharan Africa. Co-formulation of different glycoconjugates could conceivably impact the immune response to individual components. Thus, a key step is to assess the immunogenicity of the different individual vaccine components in multivalent formulations. We report herein the immunological assessment of a trivalent typhoid-iNTS conjugate vaccine formulation in rabbits measuring the induction of binding and functional antibodies.

## 2. Results

### 2.1. Immunogenicity of Monovalent and Multivalent Salmonella Glycoconjugate Vaccine Formulations in Rabbits

**Figure 2:**
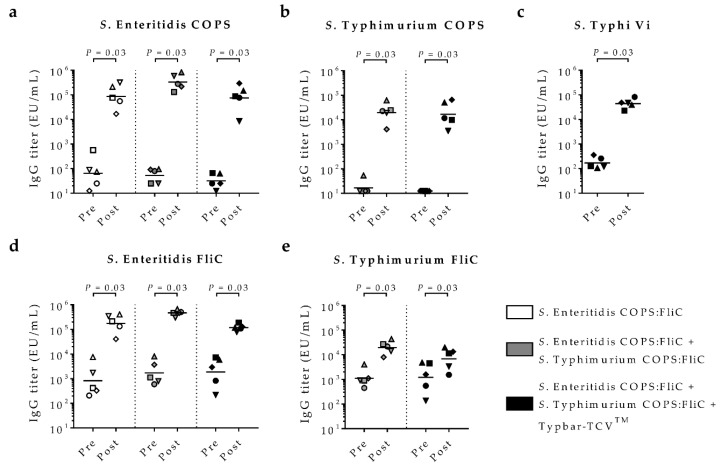
Serum IgG responses in rabbits immunized with *Salmonella* conjugate formulations. New Zealand White rabbits (*n* = 5/group) were immunized intramuscularly with monovalent *S*. Enteritidis coupled core-O polysaccharide (COPS) and phase 1 flagellin protein (FliC) (COPS:FliC) (white shapes), a bivalent nontyphoidal *Salmonella* (NTS) co-formulation (*S.* Enteritidis COPS:FliC + *S*. Typhimurium COPS:FliC, grey shapes), or a trivalent typhoid-iNTS co-formulation (*S*. Enteritidis COPS:FliC + *S*. Typhimurium COPS:FliC + *S*. Typhi Typbar-TCV™, black shapes). Baseline or day 14 post-third immunization sera were assessed for IgG titers by ELISA against (**a**) *S*. Enteritidis COPS; (**b**) *S*. Typhimurium COPS; (**c**) *S*. Typhi Vi; (**d**) *S*. Enteritidis FliC; or (**e**) *S*. Typhimurium FliC. Points represent sera from individual rabbits, bars indicate the geometric mean titer. Comparisons between groups were accomplished by 1-tailed Wilcoxon matched-pairs signed rank test, for which *p*-values are indicated.

We previously described optimized *S*. Enteritidis and *S*. Typhimurium COPS:FliC conjugates that were immunogenic and protected mice against fatal infection with the homologous serovar NTS pathogen [18,21]. Differences in the immune response to synthetic *S*. Typhimurium OPS conjugates have previously been seen between mice and rabbits [22,23]. Rabbits have also been reported previously as mounting immune responses to *Salmonella* glycoconjugate vaccine polysaccharide haptens that mediated functional antibacterial activity [23]. We first compared the anti-polysaccharide immune response to the same lot of *S*. Enteritidis COPS:FliC administered to mice or rabbits (Figure S1). Strikingly, we found that rabbits immunized with *S*. Enteritidis COPS:FliC generated uniformly higher anti-COPS IgG geometric mean titers (GMT) and levels of seroconversion compared to mice. We next compared the response to monovalent (*S*. Enteritidis COPS:FliC), bivalent (*S*. Enteritidis COPS:FliC + *S*. Typhimurium COPS:FliC) or trivalent (*S*. Enteritidis COPS:FliC + *S*. Typhimurium COPS:FliC + Typbar-TCV™) glycoconjugate formulations in rabbits. Whereas negligible anti-COPS titers were seen in pre-immune sera, robust induction of anti-COPS IgG titers were seen for sera taken 14 days after the third dose for all three formulations, with all animals seroconverting to approximately equivalent anti-polysaccharide IgG levels (Figure 2). IgG titers to *S*. Enteritidis COPS were ~10-fold higher than those induced against *S*. Typhimurium COPS. Importantly, IgG levels against *S*. Enteritidis COPS were similar between the monovalent, bivalent and trivalent formulations. IgG titers to *S*. Typhimurium COPS were also equivalent among rabbits immunized with either the bivalent or trivalent formulations. Robust induction of anti-Vi IgG was also evident among all animals immunized with the trivalent formulation, with little animal-to-animal variation. By comparison, measurable baseline anti-*S*. Enteritidis FliC titers were present in all rabbits that were significantly increased after immunization with any of the conjugate formulations (Figure 2). While baseline anti-*S.* Typhimurium FliC IgG titers were also evident among rabbits receiving the bivalent and trivalent formulations, they increased only marginally after immunization relative to pre-immune levels. Comparable COPS- and FliC-specific IgG patterns and titers were found in sera taken 44 days after the last dose (not shown).

**Figure 3:**
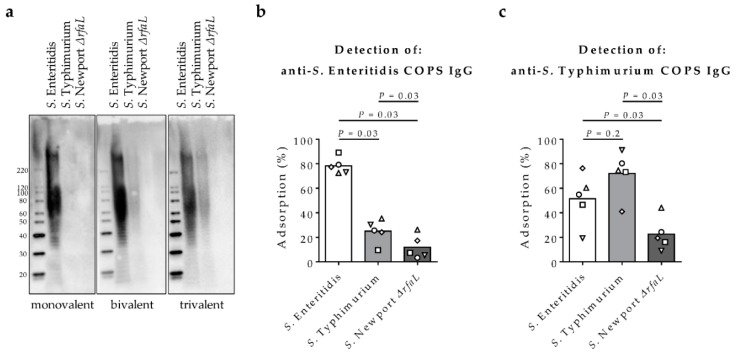
Epitope mapping of serum IgG from rabbits immunized with *Salmonella* glycoconjugates. Sera were assessed for binding by (**a**) Western blot of crude lipopolysaccharide (LPS) extracts from *S*. Enteritidis R11, *S*. Typhimurium I77, or *S*. Newport Chile 361 ∆*rfaL* using pooled rabbit sera (*n* = 5) obtained after immunization with *S*. Enteritidis COPS:FliC (monovalent), *S*. Enteritidis COPS:FliC + *S*. Typhimurium COPS:FliC (bivalent), or *S*. Enteritidis COPS:FliC + *S*. Typhimurium COPS:FliC + *S.* Typhi Typbar-TCV™ (trivalent) formulations; (**b,c**) Adsorption of anti-*S*. Enteritidis COPS IgG (**b**) and anti-*S*. Typhimurium COPS IgG (**c**) from pooled post-vaccination trivalent sera (day 14) by *S*. Enteritidis R11, *S*. Typhimurium I77, or *S*. Newport Chile 361 ∆*rfaL*. Percent adsorption was calculated relative to samples treated with bovine serum albumin (BSA). Each point indicates an individual rabbit serum sample (*n* = 5), and bars represent the mean. Groups were compared using a 1-tailed Wilcoxon matched-pairs signed rank test for which *p*-values are indicated.

*Salmonella* COPS molecules can be distinguished serologically by the presence of specific OPS epitopes (Figure 1) that are used to define serogroups but share a core polysaccharide that is conserved among all *Salmonella* serovars [1]. In order to determine the epitope preference of anti-COPS antibodies, we first assessed the pattern of binding by western blot to LPS micropreparations from whole cell lysates of *S*. Enteritidis (O:1,9,12, core polysaccharide epitopes), *S*. Typhimurium (O:1,4,[5],12, core polysaccharide epitopes), and *S*. Newport Δ*rfaL* (lacks long-chain OPS but expresses the core polysaccharide) (Table 1). We found that pooled post-vaccination serum IgG from rabbits immunized with the monovalent *S*. Enteritidis COPS:FliC bound high molecular weight *S*. Enteritidis LPS but not the heterologous LPS molecules, indicating the preponderance of antibodies against the serogroup-specific O9 epitope, but not shared OPS epitopes 1 or 12, or the core polysaccharide (Figure 3a). Incubation with serum IgG from the bivalent and trivalent formulations bound to high molecular weight *S*. Enteritidis and *S*. Typhimurium LPS. The lack of binding to low molecular weight core polysaccharide at the bottom of the gel for *S*. Newport Δ*rfaL* LPS suggested the paucity of antibodies to the core polysaccharide that is the only common COPS epitope shared between these three *Salmonella* strains. In order to further confirm the epitope preference of vaccine-induced IgG, we assessed anti-COPS titers in individual sera from rabbits immunized with the trivalent formulation after adsorption with the same isolates used for western blot analysis. We found that incubation with *S*. Enteritidis R11 was sufficient to adsorb most of the anti-*S.* Enteritidis COPS IgG titer (Figure 3b). By comparison, less adsorption of anti-*S*. Enteritidis COPS IgG was found after incubation with *S*. Typhimurium I77, and adsorption with *S*. Newport Chile 361 Δ*rfaL* was marginal. Although not statistically significant, we found that anti-*S*. Typhimurium COPS IgG was adsorbed to a greater extent with *S*. Typhimurium I77, compared to incubation with *S*. Enteritidis R11; minimal adsorption of *S*. Typhimurium anti-COPS IgG was achieved following incubation with *S*. Newport Chile 361 Δ*rfaL* cells (Figure 3c). Taken together, these results indicate that the bulk of anti-COPS IgG is directed against serogroup-specific OPS epitopes.

### 2.2. In-Vitro Functional Bactericidal Activity of Vaccine-Induced Antisera 

**Figure 4:**
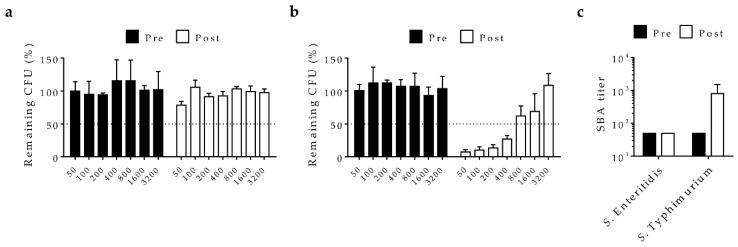
Complement-mediated bactericidal activity with sera from rabbits immunized with the trivalent typhoid-iNTS conjugate vaccine formulation. Viable colony forming units (CFU) were assessed after incubation with various dilutions of pooled pre-immune and post-vaccination trivalent sera with (**a**) *S*. Enteritidis S15 or (**b**) *S*. Typhimurium D65. Dashed line indicates 50% cutoff for titer determination. (**c**) Calculated serum bactericidal activity (SBA) titers. Bars represent mean and standard deviation from one experiment. Error bars were derived from technical replicates. Titers are representative of two independent assays.

In order to determine whether conjugate-induced antibodies could cause functional antibacterial activity, complement-mediated serum bactericidal activity (SBA) was assessed with different dilutions of pooled post-vaccination sera from rabbits immunized with the trivalent vaccine formulation compared to paired pre-immune sera (Figure 4). Incubation with pre-immune sera had no impact on *S*. Typhimurium D65 or *S*. Enteritidis S15 colony forming units (CFU) compared to incubation with complement alone. By comparison, trivalent vaccine-induced sera mediated robust bactericidal activity for *S*. Typhimurium, with a calculated SBA titer of 800. *S*. Enteritidis were only weakly killed, however, by post-vaccination sera, for which maximal killing of only ~20% of viable cells was seen at the lowest dilution tested and no increase in the endpoint titer was obtained. 

### 2.3. Functional Activity of Passively Transferred Antibodies In-Vivo 

**Figure 5:**
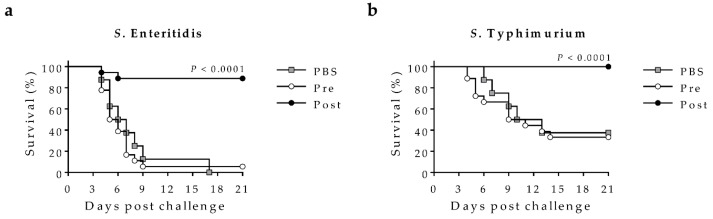
Protection against fatal iNTS infection in mice after passive transfer of trivalent vaccine-induced rabbit antisera. Mice were passively administered PBS (*n* = 8/group, grey squares) or pooled and diluted trivalent rabbit sera (*n* = 18/group), that were obtained either pre-immunization (white circles) or post-vaccination (black circles). Passively-immunized mice were then challenged with (**a**) 1 × 10^6^ CFU of *S*. Enteritidis R11 or (**b**) 1 × 10^5^ CFU of *S*. Typhimurium D65. The Kaplan–Meier survival curves for the respective pre- and post-immune sera were compared using log-rank analysis, for which P values are indicated.

Since vaccine-induced sera mediated bactericidal activity in-vitro, and we previously reported that active immunization with COPS:FliC conjugates protected mice against fatal iNTS challenge [18,21]; we sought to assess whether passive transfer of trivalent vaccine-induced sera would also protect mice against iNTS challenge. For this, we passively administered pooled pre-immune or post-trivalent vaccination sera into the peritoneal cavity of naïve mice and then challenged them intraperitoneally 3–4 h later with *S*. Enteritidis R11 or *S*. Typhimurium D65 (Figure 5). Whereas >90% of mice administered pre-immune sera or PBS succumbed to challenge with *S*. Enteritidis R11, those receiving immune sera were highly protected (Vaccine efficacy [VE] = ~88%). Similarly, while approximately 67% of mice receiving pre-immune sera or PBS succumbed to *S*. Typhimurium D65 challenge, mice administered vaccine-induced sera were fully protected (100% VE) against infection.

## 3. Discussion

Given the co-endemnicity of typhoid and iNTS in sub-Saharan Africa and the considerable overlap in the ages of peak incidence, the development and implementation of a pediatric vaccine providing coverage for both is warranted, whereby immunization during infancy may be able to provide protection throughout childhood. A prior study assessed immune responses in mice to a bivalent formulation of *S*. Typhimurium and *S*. Enteritidis COPS conjugates with CRM_197_ [28]. This is the first study to assess a trivalent NTS COPS glycoconjugate preparation that includes a Vi conjugate. We found that rabbits immunized with the trivalent formulation manifested high titers of serum IgG antibodies to all three polysaccharides. There was no impact of progressively higher valencies (i.e., monovalent, bivalent, trivalent) on antibody titers to specific polysaccharides. Surprisingly, differences were seen with respect to the patterns of antibody responses to our NTS conjugate vaccines in rabbits compared to those seen previously in mice. Antibody titers to the FliC carrier proteins were moderately lower in rabbits, but the response to *S*. Enteritidis COPS was markedly more robust [18,19,21]. This may be due to differences in the naïve B-cell repertoire, or properties of antibody paratopes recognizing different polysaccharide conformational epitopes. Rabbits often mount superior antibody responses to haptens compared to rodents [29,30]. Lindberg et al. noted that rabbits could respond immunologically to synthetic OPS *Salmonella* conjugates that failed to generate detectable antibody titers in mice [23]. Similar findings have been seen for conjugates of *Streptococcus pneumoniae* capsule type 23F, whereby immune responses in rabbits, but not mice, predicted immunogenicity in people [31]. Thus, although the mouse challenge model is widely used to assess the efficacy of *Salmonella* vaccines [32,33], rabbits may possibly be a better predictor of immune responses to *Salmonella* OPS glycoconjugates in humans.

We found that, whereas COPS:FliC induced serum antibodies mediated robust bactericidal activity against a prototype Malian *S*. Typhimurium blood isolate, these antibodies stimulated comparatively lower activity against a Malian *S*. Enteritidis blood isolate, despite manifesting higher anti-Enteritidis COPS and anti-FliC IgG titers. It is conceivable that differences in the antibody isotypes (e.g., IgM) induced after vaccination may contribute differential bactericidal activity in-vitro. A prior report found, however, that monoclonal anti-*S*. Typhimurium COPS IgG and IgM antibodies mediated comparably robust complement bactericidal activity against a Malawian *S*. Typhimurium isolate [34]. Our findings are in agreement with a prior report that documented negligible SBA killing for *S*. Enteritidis isolates from Malawi with sera from mice immunized with *S*. Enteritidis COPS conjugates with CRM_197_, but substantial SBA activity against Malawian *S*. Typhimurium isolates with sera from mice immunized with *S*. Typhimurium COPS:CRM_197_ conjugates [35]. It is thus possible that sub-Saharan *S*. Enteritidis isolates are more resistant to complement-mediated killing by anti-COPS antibodies compared to *S*. Typhimurium isolates. This should be assessed in future studies. Nevertheless, passive transfer of vaccine-induced rabbit antibodies offered significant protection against both *S*. Typhimurium and *S*. Enteritidis upon challenge of mice. The in-vivo mouse protection assay is likely a more relevant measure of the functional biological properties of vaccine-induced antibodies, however, than in-vitro bactericidal killing assays, as the protection in this model incorporates the complex environment within the host that includes different immune cell types and normal physiological levels of complement and antibody. Future studies should also assess the protection imparted after active vaccination with the trivalent formulation, as the passive protection assay used in this study may not fully recapitulate vaccine-mediated immunity to NTS bloodstream infections.

Vi conjugate vaccines are the only approved typhoid vaccine that can be given to children less than 2 years old. Foundational studies conducted in Vietnam by investigators at the US National Institutes of Health documented that conjugates of Vi with rEPA were immunogenic in young children and conferred >90% protection after 2 years of follow-up [36]. Typbar-TCV™, presently the only WHO pre-qualified Vi conjugate vaccine, was recently found to confer >80% protection against clinical typhoid in a controlled human challenge model of typhoid infection when assessed by an endpoint of fever with positive blood culture [37]. Importantly, immunization of infants and toddlers 6–23 months of age with a single dose of Tybar-TCV™ generated robust and durable antibody titers that persisted beyond 3 years [38].

Age cross-sectional studies conducted among children under 5 years old in Malawi have established a strong correlation between the titer of anti-LPS antibodies and serum bactericidal activity and diminished susceptibility to iNTS disease with *S*. Typhimurium [39]. Introduction of glycoconjugate vaccines against *Haemophilus influenzae* type b (Hib) vaccines into the routine infant immunization schedule in Mali led to a dramatic reduction in the incidence of invasive Hib disease [40]. Epidemiological modeling studies from our group have suggested that introduction of comparably efficacious broad-spectrum iNTS vaccine could have a similar impact and dramatically reduce the incidence of iNTS disease upon introduction into the routine Expanded Programme on Immunization (EPI) schedule in Africa [41]. Taken together, these prior observations provide robust support for the proposed trivalent vaccine approach that is anticipated to generate an efficacious human vaccine. Our preclinical findings herein pave the way for transition of the trivalent vaccine formulation to human clinical trials.

## 4. Materials and Methods 

### 4.1. Bacterial Strains and Growth Conditions

An overview of the different *Salmonella* strains used for this study is provided in Table 1. Growth conditions and preparation of bacteria for challenge studies and in-vitro functional assays have been described previously [18,26]. All bacteria were maintained on solid Hy-Soy agar (Teknova, Hollister, CA, USA). Bacterial cultures for in-vitro assays were grown in Hy-Soy liquid media at 250 rpm/37 °C. Fermentation cultures of bacteria for polysaccharide and protein production were grown in chemically defined media [18], that was supplemented with 0.025% guanine for CVD 1925 and CVD 1943 reagent strains, with the addition of 50 μg/mL kanamycin to maintain the pSEC10-wzzB plasmid in CVD 1925wzzB. *S*. Typhi CVD 909 was grown in Hy-Soy supplemented with 0.1 μg/mL 2,3-dihydroxybenzoic acid.

### 4.2. Polysaccharides

*S*. Enteritidis and *S*. Typhimurium COPS used for vaccine synthesis and as detection antigens in enzyme-linked immunosorbent assays (ELISA) were purified from fermentation cultures of CVD 1943 and CVD 1925wzzB as described previously by extraction with acetic acid/100 °C and purification by sequential tangential flow filtration (TFF), anion exchange chromatography and ammonium sulfate precipitation steps [18]. Removal of residual impurities in the COPS preparation was confirmed by limulus amebocyte lysate assay (LAL) for endotoxin (Charles River Laboratories, Wilmington, MA), Lowry assay for protein, and A260 nm for nucleic acid. Polysaccharide identity was confirmed by direct ELISA with anti-O9 (Genway, San Diego, CA, USA) and anti-O4 (Santa Cruz Biotechnology, Dallas, TX, USA) monoclonal antibodies. Molecular size was confirmed by high performance liquid chromatography size exclusion chromatography (HPLC-SEC) with Shodex 805/804 columns (Showa Denko, Tokyo, Japan) run in series on a Thermo, Dionex (Waltham, MA, USA) Ultimate 3000 device with UV-Diode Array Detector and refractive index (RI) at 1.0 mL/min in 10 mM PBS. Vi CPS used as ELISA antigen was purified from fermentation cultures of CVD 909. Briefly, CVD 909 was grown in Hy-Soy until stationary phase in an 8-liter fermentation culture in a Bioflo415 bioreactor (Eppendorf, Hamburg, Germany). Harvest culture was brought to 20 mM EDTA and incubated at RT for 30 min, then bacterial cells and debris were removed by centrifugation using a Sorvall RC-5B (DuPont, Wilmington, DE, USA), followed by passage through a 0.45 µm Polycap 36 TC filter (Whatman-GE, Piscataway, NJ, USA). The clarified supernatant containing Vi polysaccharide was concentrated by 30 kDa TFF (Hydrosart, Sartorius, Göttingen, Germany) and diafiltered against 10 diavolumes of 100 mM Tris/1 M NaCl/0.02 M EDTA pH 7.3 and then 10 diavolumes of DI water. The recovered retentate fraction was then brought to 2% CTAB, and incubated overnight at room temperature. The precipitated material was centrifuged at 2500× *g*/45 min/20 °C. The CTAB pellet was resuspended in 1 M CaCl_2_ and then brought to 25% ethanol and incubated 2 h at RT. The 25% ethanol precipitate was then removed by centrifugation at 2500× *g*/45 min/20 °C and the supernatant fraction then brought to 80% ethanol and incubated overnight at RT. The precipitate containing Vi was then recovered by centrifugation at 2500× *g*/45 min/20 °C and brought to 20 mM Tris/50 mM NaCl pH 7.0. The reconstituted precipitate was then bound to a HiPrep FF DEAE column (GE, Piscataway, NJ, USA) on a Bio-Rad NGC (Hercules, CA, USA) at 5 mL/min in 20 mM Tris/50 mM NaCl pH 7.0, washed with 3 column volumes (CV) of the same buffer, and eluted with 5 CV of 20 mM Tris/300 mM NaCl pH 7.0. Pooled eluate fractions were then concentrated and diafiltered into DI water with 10 kDa TFF (Hydrosart, Sartorius, Göttingen, Germany). The eluted fraction was brought to 20 mM Tris/1 mM MgCl_2_ pH 7, then incubated with 5 U/mL Benzonase (Sigma-Aldrich, St. Louis, MO, USA) for 1.5 h at 37 °C, and then 0.25 mg/mL Proteinase K (ThermoFisher, Waltham, MA, USA) for 1.5 h at 37 °C. Precipitate was removed by centrifugation at 2500× *g*/15 min/20 °C, and the supernatant was diafiltered against 10 diavolumes of PBS pH 7.4 using 50 kDa TFF (mPES, Spectrum, Rancho Dominguez, CA, USA). Purified Vi was assessed for *O*-acetylation by the method of Hestrin [42] with acetylcholine chloride standards, residual protein by Bradford assay (Biorad, Hercules, CA, USA) with bovine serum albumin (BSA, Sigma-Aldrich, St. Louis, MO, USA) standards, and concentration by dry-weight recovery after lyophilization.

### 4.3. Flagellin Proteins

FliC proteins from *S*. Enteritidis and *S*. Typhimurium were purified as described from fermentation culture supernatants of CVD 1943 and CVD 1925 respectively by cation and anion exchange membrane chromatography, and TFF [43]. The removal of endotoxin and nucleic acid was confirmed by LAL and the A280/260 nm ratio. Concentration was determined by A280 nm with the calculated extinction coefficient. Integrity and identity were confirmed by SDS-PAGE with Coomassie staining and western blot with serovar specific monoclonal antibodies as described previously [43]. 

### 4.4. Conjugate Vaccines

*S*. Enteritidis COPS:FliC lattice conjugates were generated as described by multipoint linkage between adipic acid dihydrazide (ADH, TCI, Tokyo, Japan) derivatized FliC and COPS hydroxyls activated with 1-Cyano-4-dimethylaminopyridinium tetrafluoroborate (CDAP, Sigma-Aldrich, St. Louis, MO, USA) [18,21]. *S*. Typhimurium COPS:FliC sun-type conjugates linked by a thioether bond were generated as described, by derivatization of the COPS reducing end KDO with an aminooxy-thiol linker (Fina Biosolutions, Rockville, MD, USA) followed by conjugation to FliC protein derivatized at lysine residues with *N*-γ-maleimidobutyryl-oxysuccinimide ester (GMBS, Molecular Biosciences, Boulder, CO, USA) [18]. Typbar-TCV™ is comprised of *S*. Typhi Vi conjugated to tetanus toxoid and has been described previously [38].

### 4.5. Ethics Statement 

All animal studies detailed herein were performed in facilities accredited by the Association for Assessment and Accreditation of Laboratory Animal Care and were in compliance with guidelines for animal care established by the US Department of Agriculture Animal Welfare Act, US Public Health Service policies, and US federal law. All animal experiments were in compliance with animal use protocols approved by the University of Maryland School of Medicine Institutional Animal Care and Use Committee (protocol #0715008, mice) or the Cocalico Biologicals Animal Care and Use Committee (protocol #171026CBISTD, rabbits). 

### 4.6. Animal Studies

#### 4.6.1. Rabbit Immunization

New Zealand White rabbits were immunized and bled under standard protocols at Cocalico Biologicals (Stevens, PA, USA). They were injected intramuscularly at 0, 14, and 28 days with monovalent (*S*. Enteritidis COPS:FliC), bivalent (*S*. Enteritidis COPS:FliC + *S*. Typhimurium COPS:FliC), or trivalent (*S*. Enteritidis COPS:FliC + *S*. Typhimurium COPS:FliC + Tybar-TCV™) formulated in PBS containing 5 μg of each conjugate by polysaccharide weight per dose. Sera were taken prior to the first dose and after the final immunization (days 14 and 44) and stored at −20 °C until use. 

#### 4.6.2. Mouse Immunization 

Six to eight-week old female Crl:CD-1 mice were immunized intramuscularly as described previously [18], with three doses of either PBS or 5 μg of *S*. Enteritidis COPS:FliC. Sera was taken 31 days after the last dose by retro-orbital bleed and stored at −20 °C until use.

#### 4.6.3. Passive Transfer Protection Studies

Pooled (*n* = 5) pre-immune or day 44 post-vaccination rabbit sera were heat-inactivated at 56 °C for 30 min and diluted in PBS. Six to eight-week old female Crl:CD-1 mice were injected intraperitoneally with 100 µL of sterile PBS or diluted rabbit sera. Three to 4 h later, they were challenged intraperitoneally with 1 × 10^6^ CFU of *S*. Enteritidis R11 or 1 × 10^5^ CFU of *S*. Typhimurium D65. Mice were monitored daily for 21 days after challenge recording overall health, weight loss, and mortality. Those that reached a moribund state and/or 48 h of sustained weight loss ≥20% were euthanized and recorded as having succumbed to the challenge. Vaccine efficacy was calculated by the attack rate (AR) in control and vaccinated mice as follows: VE = ((AR_controls_ − AR_vaccinated_)/AR_controls_) × 100.

### 4.7. Enzyme Linked Immunosorbent Assay (ELISA)

COPS (5 µg/mL) and FliC (5 µg/mL) were diluted in PBS and coated directly onto 96-well, medium binding microplates (Greiner Bio-One, Monroe, NC, USA) for 1 h at 37 °C. Vi used as ELISA antigen was first biotinylated as follows: Solubilized Vi was first brought to 5 mg/mL in 0.1 M MES, pH 6/0.5 M ethylene diamine with subsequent addition of 100 mg/mL 1-Ethyl-3-(3-dimethylaminopropyl)-carbodiimide (Sigma-Aldrich, St. Louis, MO, USA) in water to a final concentration of 5 mg/mL. After an overnight reaction at 4 °C, reagent was removed by exhaustive dialysis against saline. The aminated Vi was then lyophilized and resuspended to 2 mg/mL in water at which point the pH was then raised to 8 by the addition of 1/9th volume 1 M HEPES pH 8 and 1/33rd volume 0.1 M NHS-Biotin (Thermo, Waltham, MA, USA) was added. After overnight reaction at 4 °C, the solution was dialyzed extensively against saline. Nunc MaxiSorp 96-well, medium binding microplates (ThermoFisher, Waltham, MA, USA) were coated with 3 µg/mL streptavidin (Sigma, MA) diluted in sterile water at 37 °C overnight to dryness. Plates were then incubated with Vi-biotin (2 µg/mL) diluted in PBS for 3 h at 37 °C. Detection of IgG responses to COPS, FliC, and biotinylated Vi was then conducted as previously described [18] using either horseradish peroxidase (HRP)-labeled goat anti-mouse IgG or goat anti-rabbit IgG (SeraCare, Gaithersburg, MD, USA) as secondary antibodies. Endpoint titers, represented as ELISA units (EU) per mL, were defined and calculated as previously described [19]. Analyses were conducted with positive sera as plate controls with acceptance criteria of <15% variance between plates.

### 4.8. Antibody Adsorptions 

Adsorption of COPS-specific IgG from rabbit sera was conducted as previously described [44] with the following modifications: cultures of *S*. Enteritidis R11, *S*. Typhimurium D65, or *S*. Newport ∆*rfaL* were inactivated by incubation with 2% liquefied phenol (Sigma-Aldrich, St. Louis, MO) for 15 min. The bacteria were then pelleted, resuspended in 1% phenol-PBS (10% of the original volume), and stored at −80 °C until use. For the adsorption assay, phenol-inactivated bacteria were washed twice with PBS and resuspended in 3% BSA in PBS pH 7.4 (dilution buffer). Rabbit sera were diluted in dilution buffer to which either phenol-inactivated bacteria or dilution buffer (control) were added (final serum dilution was 1:500). Rabbit sera and bacteria were incubated overnight at 4 °C with tumbling rotation. The following day, bacteria with bound antibodies were pelleted, and the supernatant was assayed by ELISA for detection of remaining unbound polysaccharide-specific IgG. Percent adsorption of anti-COPS IgG refers to the titer of anti-COPS IgG after adsorption relative to the control and was calculated as follows: % adsorption = ((control–adsorbed)/control) × 100.

### 4.9. Western Blot Analysis of Lipopolysaccharide

LPS micro-preparations of *S*. Typhimurium D65, *S*. Enteritidis R11, and *S*. Newport Chile 361 *ΔrfaL* were generated as described previously [18]. LPS preparations were diluted with 100 µL of 2× Laemmli sample buffer (Bio-Rad, Hercules, CA, USA) and separated by electrophoresis on Novex 4–12% Tris-Glycine gels (Life Technologies, Carlsbad, CA, USA). The gels were wet transferred overnight at 4 °C to methanol-activated polyvinylidene difluoride (PVDF) membranes and blocked with 10% Omniblok (AmericanBio, Natick, MA, USA) + 0.1% Tween-20 (Bio-Rad, Hercules, CA, USA). The membranes were then incubated with pooled sera from rabbits immunized with monovalent, bivalent, or trivalent vaccine preparations diluted in blocking buffer (1 h, RT). Bound antibody was detected with HRP-labeled goat anti-rabbit IgG diluted in blocking buffer (1 h, RT). Membranes were again washed, incubated with Pierce ECL substrate (ThermoFisher, Waltham, MA), and visualized with the Chemi-Doc MP system (Bio-Rad, Hercules, CA).

### 4.10. Serum Bactericidal Activity (SBA) Assay

Assays were conducted as previously described [25]. Sera obtained 44 days after the final vaccine dose were heat-inactivated at 56 °C for 30 min prior to use in the assay, and serially diluted in PBS. Fifty µl of diluted antibody sample was mixed with 25 µL of baby rabbit complement (BRC) (Pel-Freez Biologicals, Rogers, AR, USA), 15 µL of PBS pH 7.2 and 10 µL of bacteria (100–350 CFU), incubated for 1 hour at 37 °C, and then plated on rich media agar to determine viable counts. Bacteria mixed with complement and saline alone were used as negative controls. Bactericidal titer was defined as the minimal dilution whereby >50% killing was observed.

### 4.11. Statistical Analyses

Statistical significance for comparisons of ELISA titers between pre-immune and post-vaccination sera and comparisons for bacterial adsorption were accomplished with one-tailed Wilcoxon matched-pairs signed rank test (α = 0.05). Mouse protection after passive transfer of rabbit sera was determined by log-rank for survival analysis, and two-tailed Fishers exact test for mortality. Calculations were performed with GraphPad Prism 6.0. *p*-values of <0.05 were considered significant. No adjustments were made for multiple comparisons.

## Figures and Tables

**Table 1:** Overview of *Salmonella* strains used in this study.

Strain	Characteristics (Use)	Reference
*S*. Enteritidis R11	Wild-type Malian blood isolate (challenge strain, epitope specificity analyses)	[24]
*S*. Enteritidis S15	Wild-type Malian blood isolate (functional antibody analyses)	[25]
*S*. Typhimurium D65	Wild-type Malian blood isolate (challenge strain, functional antibody analyses)	[24]
*S*. Typhimurium I77	Wild-type Malian blood isolate (epitope specificity analyses)	[24]
*S*. Enteritidis CVD 1943	R11 Δ*guaBA* Δ*clpP* Δ*fliD* (reagent strain for *S*. Enteritidis COPS and FliC production)	[24]
*S*. Typhimurium CVD 1925	*S*. Typhimurium I77 Δ*guaBA* Δ*clpP* Δ*fliD* Δ*fljB* (reagent strain for *S*. Typhimurium FliC production)	[24]
*S*. Typhimurium CVD 1925wzzB	*S*. Typhimurium I77 Δ*guaBA* Δ*clpP* Δ*fliD* Δ*fljB* pSEC10-wzzB (reagent strain for *S*. Typhimurium COPS production)	[18]
*S*. Typhi CVD 909	*S*. Typhi Ty2 Δ*aroCD* Δ*htrA* P_tac_*viaB* (reagent strain for Vi production)	[26]
*S*. Newport Chile 361 Δ*rfaL*	Genetically mutated *S*. Newport isolate lacking OPS (epitope specificity analyses)	[27]

## Supplementary Materials

The following are available online, Figure S1: Comparison of anti-COPS IgG responses in outbred mice and rabbits after immunization with *S*. Enteritidis COPS:FliC.
