# Supplementary material for: Immunogenicity and Induction of Functional Antibodies in Rabbits Immunized with a Trivalent Typhoid-Invasive Nontyphoidal Salmonella Glycoconjugate Formulation
Source: Molecules. 2018 Jul 17;23(7):1749. doi: 10.3390/molecules23071749 (PMC6099966; doi:10.3390/molecules23071749)
Supplement: Supplementary file 1 [file molecules-23-01749-s001.pdf]

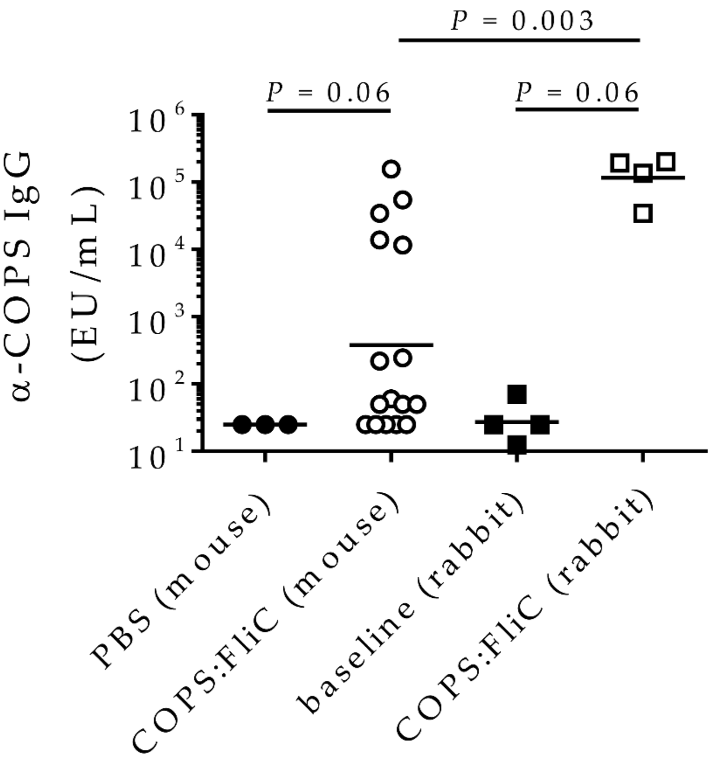

Figure S1. Comparison of anti-COPS IgG responses in outbred mice and rabbits after immunization with *S.* Enteritidis COPS:FliC. Mice were immunized three times with either PBS (n = 3) or COPS:FliC formulated with monophosphoryl lipid A (n = 16). Alternatively, rabbits were immunized three times with COPS:FliC without adjuvant (n = 4). Anti-COPS IgG titers in post-vaccination sera were assessed on day 31 (mice) and day 14 (rabbits). Points represent individual serum samples. Lines represent the geometric mean titer, which was compared using a 1-tailed Mann-Whitney U test (mice) or 1-tailed Wilcoxon matched-pairs signed rank test (rabbits). Comparisons between mice and rabbit post-immune sera were by 2-tailed Mann-Whitney U test. P-values for indicated comparisons are displayed in the figure.
